# Abdominal Distension in an Elderly Man after Presumed Vertical Transmission of Chagas Disease

**DOI:** 10.4269/ajtmh.18-0764

**Published:** 2019-04

**Authors:** Eliza Cricco-Lizza, Montreh Tavakkoli, Justin R. Kingery

**Affiliations:** Department of Medicine, New York-Presbyterian Hospital/Weill Cornell Medical Center, New York City, New York

**Figure 1.**
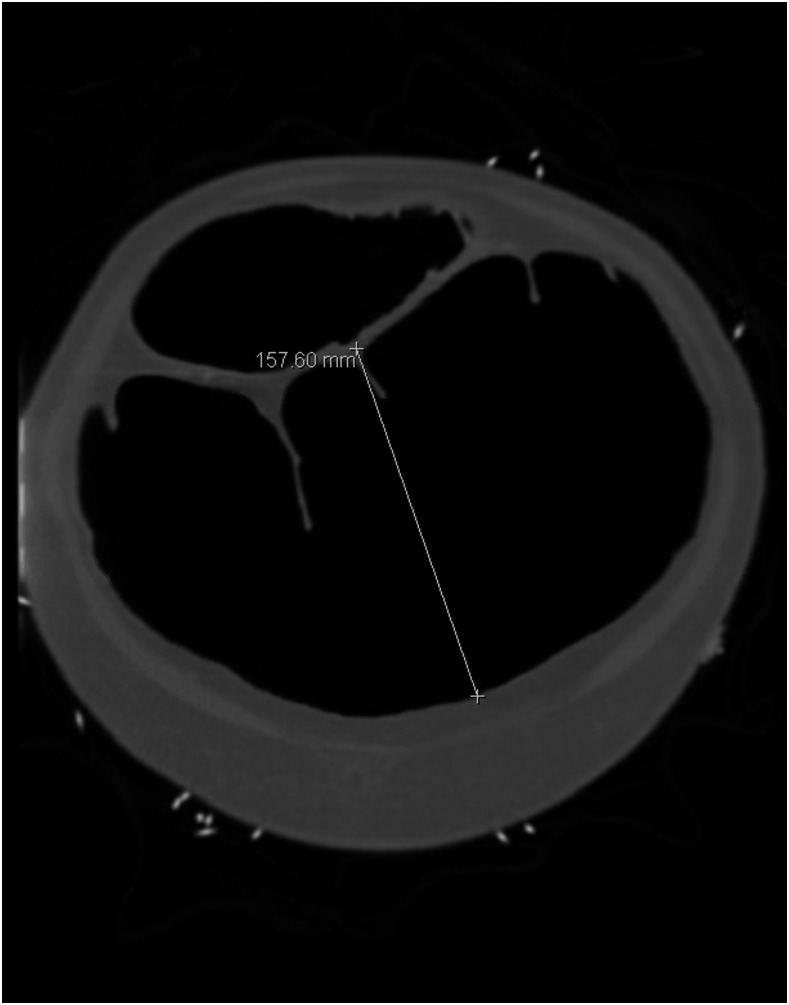
Computed tomographic study of the abdomen and pelvis in the coronal plane revealing a 16-cm dilatation of the transverse colon.

**Figure 2.**
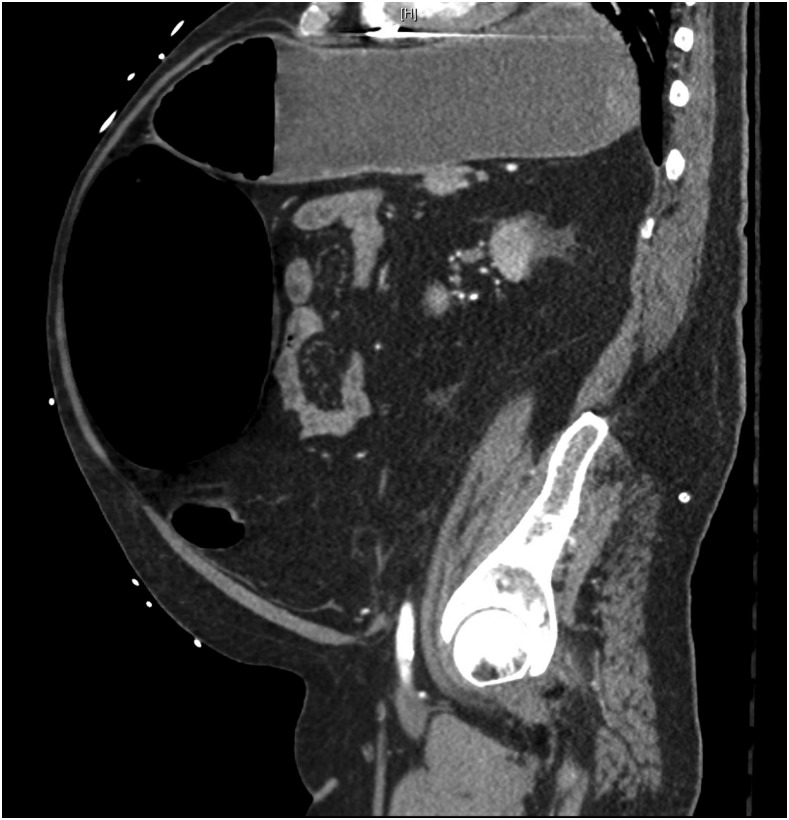
Computed tomographic study of the abdomen and pelvis in the sagittal plane revealing distention of the stomach, duodenum, and transverse colon.

**Figure 3.**
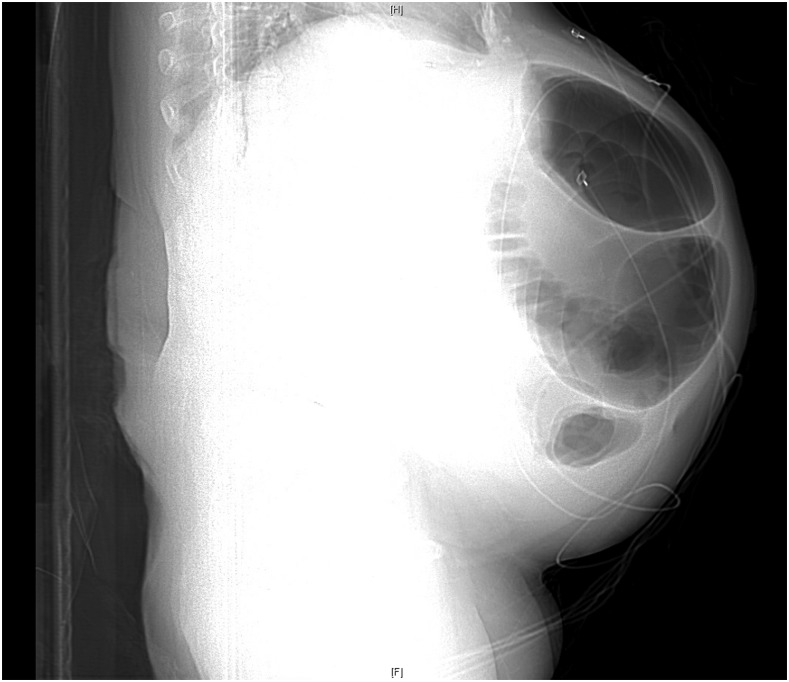
Scout view of the abdomen (lateral projection) on computed tomographic study of the abdomen and pelvis.

Chagas is a parasitic disease caused by *Trypanosoma cruzi*. It is commonly asymptomatic in the acute phase. Chronically, it can lead to heart block, cardiomyopathy, and/or megaesophagus/colon.^1,2^ Vertical transmission occurs in 1–10% of births and is under-recognized and rarely screened.^3^ In a survey of obstetrician-gynecologists in the United States, only 8% knew the risk of congenital infection and 78% never considered a diagnosis of Chagas disease among patients from endemic countries.^3^ We present the case of an 81-year-old man from the United States with a history of complete heart block who presented with chronic abdominal distention. Abdominal imaging revealed distention of the stomach and duodenum, and 16 cm enlargement of the transverse colon (5.7 SD above the mean for Chagas megacolon) (Figures 1–3).^2^ *Trypanosoma cruzi* IgG was positive by ELISA, and IgM and polymerase chain reaction were negative. The patient reported travel limited to Cuba, France, and the Democratic Republic of Congo, where no documented cases of transmission have been observed.^1^ His mother, however, originated from South America, making vertical transmission the likely source of his infection. Given effective treatment strategies with early diagnosis, raising awareness and screening for vertical transmission could thwart preventable complications of this disease.^3^
